# Characterization of the complete chloroplast genome of *Elaeagnus pungens* (elaeagnaceae) and phylogeny within elaeagnaceae

**DOI:** 10.1080/23802359.2022.2090291

**Published:** 2022-07-04

**Authors:** Yin Lu, Qing Ma, Xiaolu Xu, Zhimin Wang, Ivanets Andrie, Tatsiana Savitskaya, Seyit Yuzuak

**Affiliations:** a College of Biology and Environmental Engineering, Zhejiang Shuren University, Hangzhou, Zhejiang, China; b Institute of General and Inorganic Chemistry, National Academy of Sciences of Belarus, Minsk, Belarus; c Department of Chemistry, National University of Belarus, Minsk, Belarus; d Department of Molecular Biology and Genetic, Mehmet Akif Ersoy University, Burdur, Turkey

**Keywords:** *Elaeagnus pungens*, chloroplast genome, phylogeny, Elaeagnaceae

## Abstract

*Elaeagnus pungens* is an evergreen shrub with high medicinal values. In this study, the complete chloroplast (cp) genome of *E. pungens* was characterized. The chloroplast genome of *E. pungens* is 152,218 bp in length, consisting of two 25,876 bp inverted repeats, 18,231 bp small single copy region, and 82,235 bp large single copy region. The chloroplast genome contains 113 unique genes, including 79 protein-coding genes, 30 tRNA genes, and 4 rRNA genes. Phylogenomic analysis revealed that *E. pungens* and species from *Elaeagnus* formed a monophyletic clade sister to the clade consisting of species from *Hippophae*.

Belonging to the Elaeagnaceae family, *Elaeagnus* Linn. 1754 is a genus consisting of ∼60 species distributed in different continents worldwide (Heywood et al. 2007). *Elaeagnus pungens* Thunb. 1784, an evergreen shrub native to Japan and South China, is a member of the *Elaeagnus* genus. The leaves of *E. pungens* have been well-recognized as a traditional medicinal material. They are mainly used in prescriptions for the treatment of respiratory and digestive system diseases such as cough, tracheitis, hematemesis, dysentery, and enteritis. Previous studies showed that water extracts of *E. pungens* leaves were rich in flavonoids with antiasthmatic, antitussive, and expectorant effects (Ge et al. 2009). However, the current studies of *E. pungens* have been limited to its chemical component, pharmacological effect, and cultivation strategy. The genetic background of *E. pungens* has been largely ignored. In this study, we characterized the complete chloroplast genome of *E. pungens* to provide genomic and genetic resources for future molecular analysis.

Total genomic DNA was extracted from silica-dried leaves of one *E. pungens* plant individual cultivated in Hangzhou, Zhejiang Province, China (30.26 N, 120.12 E) using modified CTAB method (Doyle and Doyle, 1987). Collection of the plant material is in compliance with Plant Material Collection Guidelines of Zhejiang Shuren University. The study did not involve any endangered or protected species. The sample collection site is neither privately owned nor protected for which no specific permission was required. The voucher specimen (No. MQ20-20008) was deposited in the herbarium of Zhejiang Shuren University (Contact: Qing Ma, maqing90@live.cn). The chloroplast genome was first sequenced using the Illumina Hiseq Platform (Illumina, San Diego, CA) at BGI (Shenzhen, Guangdong, China). The obtained raw reads were preliminarily screened to remove adaptor sequences and low-quality sequences. Next, de novo assembly of filtered paired-end reads were conducted using the GetOrganelle software (Jin et al. 2020) and SPAdes 3.10.1 (Bankevich et al. 2012). Genome annotation was performed with Geneious R11 (https://www.geneious.com) using the chloroplast genome of *Elaeagnus umbellata* (GenBank accession number: LC522506) as the reference. The putative starts, stops, and boundaries between exons and introns were manually checked by comparison with homologues genes of published Elaeagnaceae species. The tRNA genes were verified using tRNAscan-SE v1.21 (Schattner et al. 2005). The complete chloroplast genome sequence of *E. pungens* after annotation was deposited to GeneBank under the accession No. MW145133.

The chloroplast genome size of *E. pungens* is 152,218 bp, which included two 25,876 bp inverted repeats (IRs), a 18,231 bp small single copy (SSC) region, and a 82,235 bp large single copy (LSC) region. The guanine and cytosine (GC) contents of the LSC, SSC, and IR regions were 34.9%, 30.6%, and 42.7%, respectively. The whole chloroplast genome contains a total of 113 unique genes, including 79 protein-coding genes, 30 tRNA genes, and 4 rRNA genes. Five protein-coding (*ndhB*, *rps7*, *rpl2*, *rpl23,* and *ycf2*), eight tRNA (*trnA-*UGC, *trnH-*GUG, *trnI-*CAU, *trnI-*GAU, *trnL-*CAA, *trnN-*GUU, *trnR-*ACG, and *trnV-*GAC), and four rRNA genes (*rrn4.5*, *rrn5*, *rrn16,* and *rrn23*) are duplicated and located in the IR regions.

The evolutionary relationships of *E. pungens* and other 13 related species from Elaeagnaceae were analyzed using the whole chloroplast genome sequences. One species, *Barbeya oleoides* from Barbeyaceae (NC_040984) was selected as the outgroup according to the results of Choi et al. (2015). Sequence alignment was conducted using MAFFT version 7.0 (Katoh et al., 2017) and phylogenomic analysis was conducted using the maximum likelihood (ML) and Bayesian inference (BI) methods with the RAxML-HPC v8.1.11 and MrBayes v3.2.3 online tools implemented on the CIPRES Science Gateway (http://www.phylo.org/; Ronquist and Huelsenbeck 2003; Miller et al. 2010; Stamatakis, 2014). Both the ML and BI analyses were performed using GTR + I + G as the best-fit nucleotide substitution model. All the 13 species from Elaeagnaceae form one clade with full support on all the nodes. The nine *Elaeagnus* species formed a monophyletic clade sister to the clade consisting of four *Hippophae* species. Both the *Elaeagnus* and *Hippophae* clades were fully supported by ML and Bayesian analyses (Figure 1). The topology of phylogenetic tree obtained in this study is largely consistent with previous phylogenetic study of Elaeagnaceae based on ITS sequences of nuclear rDNA, which showed that *Elaeagnus glabra* was clustered with *Elaeagnus macrophylla*, while *Elaeagnus multiflora* was clustered with *Elaeagnus umbellate* (Son et al. 2014). In the future, more species and more samples from each species are necessary to fully resolve the phylogeny of Elaeagnaceae.

**Figure 1.**
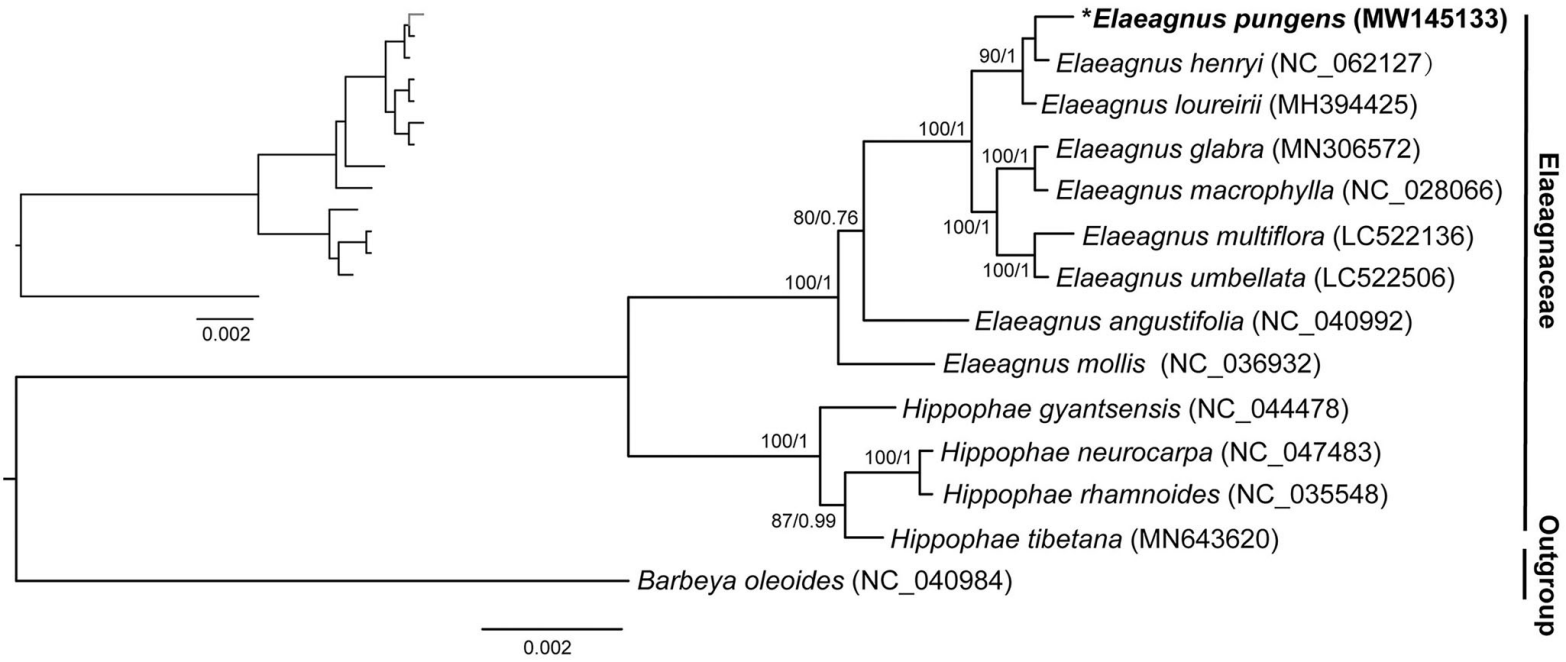
Phylogenetic tree reconstruction of *Elaeagnus* and *Hippophae* in Elaeagnaceae using ML based on whole chloroplast genome sequences. The newly sequenced species in the study is indicated using bold letter and an asterisk. GenBank accession numbers of all the chloroplast genome used for phylogenomic analysis are indicated in the brackets. Numbers above the branches represent bootstrap values from maximum likelihood analyses (before slash) and posterior probabilities from Bayesian inference (after slash), respectively.

## Data Availability

The genome sequence data that support the findings of this study are openly available in GenBank of NCBI at (https://www.ncbi.nlm.nih.gov/) under the accession no. MW145133. The associated BioProject, Bio-Sample, and SRA numbers are PRJNA669307, SAMN16450966, and SRR16146945.
